# Highly carbapenem-resistant *Achromobacter xylosoxidans* harboring *bla*_NDM-1_ in Myanmar

**DOI:** 10.1128/spectrum.00080-25

**Published:** 2025-05-19

**Authors:** Maiko Kirikae, Satoshi Oshiro, Satomi Takei, Naeko Mizutani, Atsuo Itakura, Pan Ei Soe, Thi Thi Htoon, Swe Setk, Htay Htay Tin, Teruo Kirikae, Tatsuya Tada

**Affiliations:** 1 Department of Obstetrics and Gynecology, Juntendo University Faculty of Medicine, Tokyo, Japan; 2 Juntendo Advanced Research Institute for Health Science, Juntendo University, Tokyo, Japan; 3 Department of Clinical Laboratory Medicine, Juntendo University Graduate School of Medicine, Tokyo, Japan; 4 National Health Laboratory, Yangon, Myanmar; 5 Department of Microbiology, Juntendo University Graduate School of Medicine, Tokyo, Japan; 6 Department of Clinical Laboratory Technology, Juntendo University Faculty of Medical Science, Chiba, Japan; JMI Laboratories, North Liberty, Iowa, USA

**Keywords:** *Achromobacter xylosoxidans*, multidrug resistance, carbapenemase, *bla*_NDM-1_

## Abstract

*Achromobacter xylosoxidans* is a multidrug-resistant, non-glucose-fermenting, gram-negative bacterium with intrinsic resistance to many antimicrobial agents. Between 2016 and 2017, five *A. xylosoxidans* isolates were obtained from five patients at three hospitals in Myanmar. Minimum inhibitory concentrations (MICs) against various antimicrobial agents were determined using the microdilution method. Whole genome sequencing was performed with the MiSeq and MinION platforms. Resistance genes and their surrounding structures were identified and compared. All five isolates were resistant to amikacin and aztreonam. Among them, one isolate, MyNCGM749, was resistant to imipenem and meropenem with MICs of 256 µg/mL and amikacin with MIC of >512 µg/mL but intermediate to ciprofloxacin with MIC of 2 µg/mL. The isolate carried *bla*_NDM-1_ encoding metallo-β-lactamase, *bla*_PSE-1_ encoding extended-spectrum-β-lactamase, and *bla*_OXA-114_ (encoding intrinsic -β-lactamase present in *A. xylosoxidans*), along with five aminoglycoside modification encoding genes including *aac(6')-Ib*, *aph (6)-Id*, *aph(3'')-Ib*, *ant(4')-Iib*, and *aph(3')-VI* on its chromosome. The genetic structure surrounding *bla*_NDM-1_ contained four IS*91* elements identical to those found in carbapenem-resistant *Pseudomonas asiatica* isolates in Myanmar. This is the first report of *A. xylosoxidans* in Myanmar. Although *A. xylosoxidans* harboring *bla*_NDM-1_ has been reported in a single strain from India, its genomic details have not been previously described. This study indicates that the *bla*_NDM-1_-containing structure flanked by IS*91* is spreading among gram-negative, non-glucose-fermenting bacteria in Myanmar and neighboring countries.

**IMPORTANCE:**

*Achromobacter* species were originally environmental organisms that became opportunistic pathogens with multidrug resistance. *Achromobacter xylosoxidans* is associated with nosocomially acquired infections affecting multiple organ systems, including the respiratory and urinary tracts, and, less commonly, the cardiovascular and central nervous systems. To date, carbapenem-resistant *A. xylosoxidans* carrying carbapenemase-encoding genes has been reported in several countries, including Greece, India, Italy, Japan, Korea, Libya, and the Netherlands. In this molecular epidemiological study on *A. xylosoxidans* in Myanmar, we identified the genomic structure surrounding *bla*_NDM-1_, flanked by IS*91*. This structure may facilitate the spread of non-glucose-fermenting gram-negative bacteria, such as *Achromobacter*, *Pseudomonas,* and *Stenotrophomonas* species, in Asian countries.

## INTRODUCTION

*Achromobacter xylosoxidans* (synonym: *Alcaligenes xylosoxidans*) is a gram-negative, aerobic, non-spore-forming, and motile rod with peritrichous flagella (1). It is the type species of the genus *Achromobacter*, which currently consists of 22 species (https://lpsn.dsmz.de/search?word=achromobacter). *A. xylosoxidans* is well-known for causing respiratory tract infections in cystic fibrosis patients (2) and a wide range of infections in immunocompromised individuals. These include catheter-related infections in dialysis patients (3), bloodstream infections in patients with high-risk malignancies (4) and premature infants (5), and urinary tract infections in patients with malignancies or urological abnormalities (6). *A. xylosoxidans* is commonly found in wet environments, including hospitals, households, and outdoor settings (7).

*A. xylosoxidans* exhibits intrinsic resistance to several antibiotics, including most aminoglycosides, aztreonam, cephalosporins, fluoroquinolones, nalidixic acid, penicillins (8), and polymyxin/colistin (9). These resistance mechanisms are partly attributed to chromosomally encoded multidrug efflux pumps, such as AxyABM (10) and AxyXY-OprZ (11). AxyABM is involved in resistance to cephalosporins (except cefepime), aztreonam, nalidixic acid, fluoroquinolones, and chloramphenicol (CHL) (10); and AxyXY-OprZ is involved in resistance to aminoglycosides (tobramycin, amikacin, and gentamicin) (11). These resistance mechanisms are also attributed to chromosomally encoded class D β-lactamase, OXA-114, which has a narrow-spectrum hydrolysis profile, although it includes imipenem, at a low level (12). Resistance to polymyxin/colistin is associated with unusual lipopolysaccharide lipid A moiety, including the loss of a phosphate group and the presence of penta-acylated fatty chains (9). Genome analysis of the *A. xylosoxidans* type strain hypothesized the presence of 50 drug resistance-associated genes, including five β-lactamase genes and 17 efflux pump genes (8).

*A. xyloxoxidans* can acquire carbapenem resistance through horizontal gene transfer. Previous studies have reported clinical isolates harboring *bla*_IMPs_ genes in Japan (13); *bla*_NDM-1_ in India (14); *bla*_VIMs_ genes in Italy (15), Greece (16), and in Korea (17); and *bla*_AXC_ in the Netherlands (18). Additionally, *bla*_TMB-1_ was detected in an isolate from a hospital environment in Libya (19). However, these reports provide insufficient detail about the genomic structures and surrounding carbapenemase-encoding genes. In this study, we analyzed the genetic and epidemiological characteristics of five clinical isolates of *A. xylosoxidans* from Myanmar.

## MATERIALS AND METHODS

### Bacterial strains and drug susceptibility testing

Between January and December 2016, five *A. xylosoxidans* isolates were obtained from five patients at three hospitals in Myanmar: two isolates from hospital A, two from hospital B, and one from hospital C. Bacteria identification was performed using the VITEK 2 system (bioMérieux, Marcy l’ Etoile, France), and the identities were confirmed by sequencing the 16S rRNA gene. The isolates were obtained from various clinical samples: two from sputum, one from blood, one from urine, and one from a wound. Minimum inhibitory concentrations (MICs) were determined using the broth microdilution method, following the guidelines of the Clinical and Laboratory Standards Institute (20).

### Whole genome sequencing and identification of drug-resistant genes

Genomic DNA from all isolates was extracted using DNeasy Blood and Tissue kits (Qiagen, Tokyo, Japan) and sequenced using a short-read sequencer, MiSeq (Illumina, San Diego, CA, USA). The raw sequencing reads were assembled using CLC Genomic Workbench version 10.0.1 (CLC bio, Aarhus, Denmark). For the isolate exhibiting high carbapenem resistance, genomic DNA was additionally extracted using QIAGEN Genomic-tip 20/G and the Genomic DNA Buffer Set (Qiagen) and sequenced using the MinION platform (Oxford Nanopore Technologies, Oxford, UK). The quality of the sequencing reads was evaluated by the base calling (average rates of Q30: 85.1%), and the coverage depths of each genome were equal or more than 100-fold. The raw reads from MiSeq and MinION were assembled using Unicycler v. 0.4.7. The evaluation of the hybrid assemblies was conducted by BUSCO v. 5.8.0. Drug resistance genes were identified using ResFinder v. 4.6.0 (http://genepi.food.dtu.dk/resfinder). Resistance to fluoroquinolones was assessed for mutations in the quinolone resistance-determining regions (QRDRs) of the *gyrA* and *parC* genes, which encode DNA gyrase and topoisomerase IV, respectively, using CLC Genomic Workbench v. 10.0.1 (21). The genomic environment surrounding *bla*_NDM-1_ was determined and visualized using the Basic Local Alignment Search Tool (https://blast.ncbi.nlm.nih.gov/Blast.cgi?PROGRAM=blastn&PAGE_TYPE=BlastSearch&LINK_LOC=blasthome) and *in silico* Molecular Cloning: Genomics Edition (*In Silico* Biology, Inc, Yokohama, Japan).

### dDDH and ANI values of *Achromobacter* strains

Genomic sequences from 46 *A*. *xylosoxidans* strains derived from various countries, including Australia, Argentina, Canada, China, the Czech Republic, France, Germany, India, Japan, Myanmar (in this study), Nigeria, Oman, Russia, Serbia, Taiwan, Thailand, the United Kingdom, and the United States, were retrieved from GenBank (https://www.ncbi.nlm.nih.gov/nuccore) (Table S1). Digital DNA-DNA hybridization (dDDH) and Average Nucleotide Identity (ANI) values of the 46 strains were calculated against the *A. xylosoxidans* type strain. Of them, the strains with 70% or less dDDH values or 95% or less ANI values were analyzed to determine the closest species using TYPE (STRAIN) GENOME SERVER server (Type Strain Genome Server). dDDH and ANI values against the closest type strains were calculated.

### Phylogenetic analysis based on single nucleotide polymorphisms

Genomic sequences of the 46 strains tested were aligned against the genome of the *A. xylosoxidans* type strain (GenBank accession no. NZ_LN831029). A phylogenetic tree was constructed using kSNP4.0 (22). Among *A. xylosoxidans* isolates of which the complete genome sequences were registered in GenBank, the numbers of SNPs were calculated, and the SNP matrix was calculated using snp-dists v. 0.8.2.

## RESULTS

### Drug susceptibilities of *A. xylosoxdans* isolates

As shown in Table 1, all tested isolates were resistant to amikacin, aztreonam, and cefepime, while they were susceptible or intermediately resistant to ciprofloxacin and minocycline. Among the five isolates, three were resistant to both CHL and trimethoprim-sulfamethoxazole. Two isolates exhibited high resistance to ceftazidime, with MICs of >256 µg/mL, and one isolate demonstrated high resistance to both imipenem and meropenem, with MICs of 256 µg/mL. Additionally, all isolates exhibited relatively high-level resistance to colistin, with MICs of >2 µg/mL.

**TABLE 1:** Characteristics of the five *A. xylosoxidans* isolates in Myanmar, including their antimicrobial resistance profiles and drug resistance factors

Isolates	Hospital	MIC (µg/mL)^*a*^												β-lactamase-encoding genes	Aminoglycoside-modifying enzymes encoding genes	Tetracycline resistance genes	SXT resistance gene	CHL resistance genes
		AMK	AZT	CAZ	CHL	CIP	CST	FEP	IPM	MEM	MIN	SXT	TIM					
MyNCGM70	A	128	256	4	256	2	8	64	4	1	8	64/1,216	256/2	*bla*_AXC-1_, *bla*_OXA-114_, *bla*_PSE-1_	*aac(6')-Ib, aph (6)-Id, aph(3'')-Ib*	*tet(G*)	*dfrA15*	*cmx*, *floR*
MyNCGM121	B	>512	64	8	8	8	16	128	4	0.25	8	0.063/1.1	2/2	*bla*_AXC-1_, *bla*_OXA-114_	Not detected	Not detected	Not detected	Not detected
MyNCGM152	B	256	>512	256	512	2	4	128	2	0.5	2	64/1,216	32/2	*bla*_OXA-21_, *bla*_OXA-114_	*aac(6')-Ib, aph (6)-Id, aph(3'')-Ib*	Not detected	*dfrA1*	*cmx*, *floR*
MyNCGM683	C	128	>512	8	16	1	2	32	4	0.25	4	0.032/0.6	4/2	*bla*_OXA-114_, *bla*_TEM-1_	*aac(6')-Ib-cr*	Not detected	Not detected	Not detected
MyNCGM749	C	>512	>512	>512	32	2	8	>512	256	256	2	64/1,216	>512/2	*bla*_NDM-1_, *bla*_OXA-114_, *bla*_PSE-1_	*aac(6')-Ib, ant(4')-Iib, aph (6)-Id, aph(3'')-Ib, aph(3')-VI*	*tet(B*)	Not detected	Not detected

^*a*^ AMK; amikacin, AZT; aztreonam, CAZ; ceftazidime, CHL; chloramphenicol, CIP; ciprofloxacin, CST; colistin, FEP; cefepime, IPM; imipenem, MEM; meropenem, MIN; minocycline, SXT; trimethoprim-sulfamethoxazole, and TIM; ticarcillin-clavulanic acid.

### Drug-resistant genes of *A. xylosoxidans*

Table 1 summarizes the drug resistance-associated genes and mutations identified in the isolates. All tested isolates harbored *bla*_OXA-114_, an intrinsic β-lactam resistance gene of *A. xylosoxidans*. Among them, two isolates carried *bla*_AXC-1_, a carbapenemase-encoding gene specific to the *Achromobacter* genus, and one isolate harbored *bla*_NDM-1_. Four of the five isolates carried aminoglycoside-modifying enzyme genes, such as *aac(6′)-Ib* or *aac(6′)-Ib-cr*. Additionally, two isolates harbored *tet(G*) or *tet(B*) associated with tetracycline resistance, two harbored *dfrA15* or *dfrA1* associated with trimethoprim-sulfamethoxazole (SXT) resistance, and two harbored *cxm* or *floR* associated with CHL resistance. In the QRDRs, all isolates exhibited specific point mutations: S83Q in GyrA and S80Q and E84D in ParC.

### Genetic environments surrounding *bla*_NDM-1_

**Fig 1:**
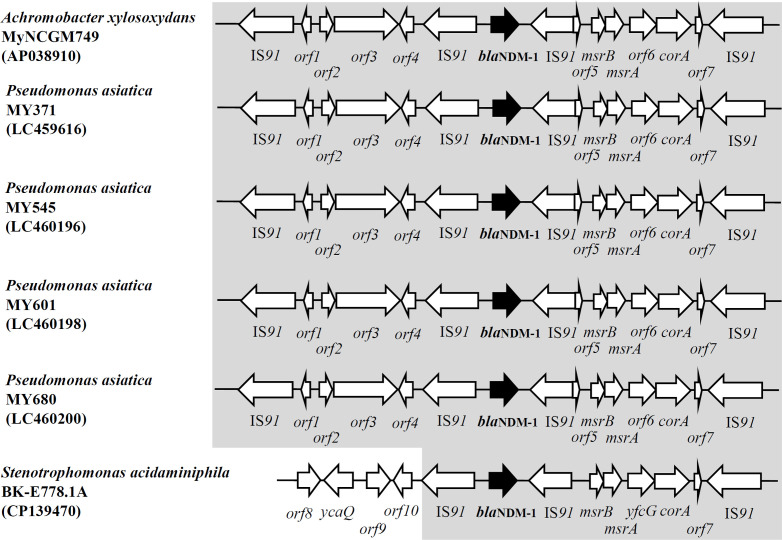
Genomic environments surrounding *bla*_NDM-1_ in *A. xylosoxidans* MyNCGM152. The genetic environment of *bla*_NDM-1_ in MyNCGM749 closely resembled those observed in clinical isolates of *P. asiatica* from Myanmar and an environmental isolate of *S. acidaminiphila* from Bangladesh. Abbreviations: *orf1*: gene encoding hypothetical protein, *orf2*: gene encoding CPBP family intramembrane glutamic endopeptidase, *orf3*: gene encoding ABC transporter ATP-binding protein, *orf4*: gene encoding hypothetical protein, *orf5*: gene encoding hypothetical protein, *orf6*: gene encoding glutathione S-transferase N-terminal domain-containing protein, *orf7*: gene encoding hypothetical protein, *orf8*: gene encoding DNA repair ATPase, *orf9*: gene encoding nucleotidyltransferase domain-containing protein, and *orf10*: gene encoding multicopper oxidase domain-containing protein.

As shown in Fig. 1, the genomic structure surrounding *bla*_NDM-1_ consisted of the arrangement IS*91-orf1-orf2-orf3-orf4-*IS*91-bla*_NDM-1_*-*IS*91-orf5-msrB-msrA-orf6-corA-orf7-*IS*91*, which included four IS*91* elements. This structure was identical to that found in *Pseudomonas asiatica* isolates (MY371, MY545, MY601, and MY680) from Yangon and Mandalay, Myanmar, in 2016 and 2017 (GenBank accession numbers LC459616, LC460196, LC460198, and LC460200). Additionally, a partial structure (*orf8-ycaQ-orf9-orf10-*IS*91-bla*_NDM-1_*-*IS*91-msrB-msrA-yfcG-corA-orf7-*IS*91*) was identical to that reported in *Stenotrophomonas acidaminiphila* BK-E778.1A, isolated from a hospital surface in Bangladesh in 2016 (GenBank accession no. CP139470).

### Phylogenetic analysis and SNP-matrix of *A. xylosoxidans*

Phylogenetic analysis based on 46 genomes (five from Myanmar and 41 from other countries) revealed two clades, Clade A and Clade B (Fig. S1). However, ANI and dDDH analyses indicated that FDAARGOS_147 and six isolates in Clade B did not belong to *A. xylosoxidans* (Table S2a). Of them, two were identified as *A. aegrifaciens* using their dDDH and ANI values against the type strain of the closest species, whereas the other five strains may not be known species since their dDDH and ANI values were less than 70% and 95%, respectively (Table S2b).

**Fig 2:**
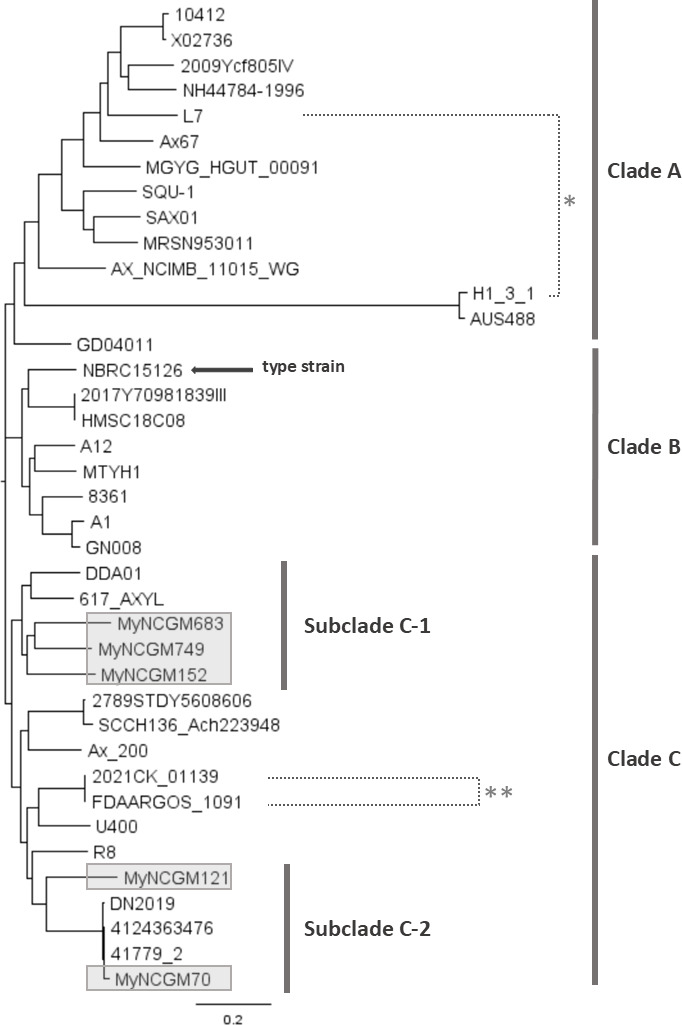
Molecular phylogenetic tree of *A. xylosoxidans* strains based on whole genome sequences. The tree includes 39 strains collected from several countries, including Australia, Argentina, Canada, China, France, Germany, India, Japan, Nigeria, Oman, Russia, Serbia, Thailand, the United Kingdom, and the United States. The five strains tested in this study, obtained from Myanmar, are highlighted in gray. Asterisk (*) indicates a pair of strains with the furthest distance. Asterisks (**) indicate with the closest distance.

Therefore, a revised phylogenetic tree was constructed using the remaining 39 genomes (Fig. 2). This updated analysis revealed three distinct clades: Clade A, Clade B, and Clade C (Fig. 2). The five isolates tested in this study (highlighted in gray) belonged to Clade C, which was further divided into two subclades, C-1 and C-2. In Subclade C-1, MyNCGM152 was isolated from hospital B in Yangon, while MyNCGM683 and MyNCGM749 were from hospital C in Mandalay. In Subclade C-2, MyNCGM70 and MyNCGM121 were isolated from hospitals A and C, respectively, in Yangon. The genetic backgrounds of the Subclade C-1 isolates (MyNCGM152, MyNCGM683, and MyNCGM749) were closely related to *Achromobacter* isolates DDA01 from Nigeria in 2022 and 617_AXYL from the United States (year unknown). Conversely, the Subclade C-2 isolate MyNCGM70 shared high similarity with *Achromobacter* strains: DN2019 from France in 2019, 4124363476 from Canada in 2014, and 41779_2 from Russia in 2020 (Fig. 2). It is unclear that the five isolates from Myanmar exhibit similar synteny to each other because four of the five isolates, except for MyNCGM749, were sequenced by a short-read sequencer.

As shown in Table S3, the number of SNPs between the strains 2021CK_01139 and FDAARGOS_1091 was 126, which was the lowest, and these isolates were closely related to each other in the phylogenetic tree (asterisks** in Fig. 2), whereas the number between the strains H1_3_1 and L1 was 78,664, which was the highest, and these isolates were far from each other in the phylogenetic tree (asterisk* in Fig. 2). Among the five isolates in Myanmar, the numbers of SNPs among MyNCGM152, MyNCGM683, and MyNCGM749 ranged from 19,512 to 21,091, and they belonged to Subclade C-1 in the phylogenetic tree (Fig. 2), whereas the number of SNPs between MyNCGM70 and MyNCGM121 was 20,294 (Table S3), and they belonged to Subclade C-2 (Fig. 2). Among five strains in Subclade C-2, four strains, including 4124363476 from Canada, 41779_2 from Russia, DN2019 from France, and MyNCGM70 from Myanmar, were genetically close to each other with the number of SNPs from 154 to 1,049. Collectively, these data indicate the numbers of SNPs were correlated with the genetic distances in the phylogenetic tree.

## DISCUSSION

Non-glucose-fermenting gram-negative bacteria harboring *bla*_NDM-1_, including *Achromobacter* spp. *and Pseudomonas* spp., may have spread in Myanmar and neighboring countries. In our previous study, several *P. asiatica* clinical isolates with an identical genomic structure surrounding *bla*_NDM-1_ as observed in *A. xylosoxidans* MyNCGM749 were isolated from two different regions, Mandalay and Yangon, in Myanmar (23). Additionally, *S. acidaminiphila* with a partially identical genomic structure surrounding *bla*_NDM-1_ was detected in Bangladesh (GenBank accession no. CP139470). A recent study reported an isolate of carbapenem-resistant *A. xylosoxidans* harboring *bla*_NDM-1_ in India, although its genetic environment was not detailed (14).

Drug-resistant *A. xylosoxidans* has emerged as an important nosocomial pathogen. This bacterium can easily spread in hospitals despite being a relatively low-virulence gram-negative pathogen compared to others. Its low virulence is attributed to its unusual lipopolysaccharide lipid A moiety, characterized by monophosphate and penta-acylated fatty chains with low endotoxic activity (24, 25). Furthermore, *A. xylosoxidans* exhibits intrinsic resistance to multiple classes of antibiotics, including aminoglycosides, β-lactams, CHL, quinolones (8), and polymyxins/colistin (9), which facilitates its survival under selective pressure in hospital settings. The isolate, MyNCGM121, was highly resistant to amikacin; nonetheless, it did not harbor any genes encoding aminoglycoside modification enzymes (Table 1). This aminoglycoside resistance may be explained by the presence of the intrinsic efflux pump, AxyXY-OprZ, in *A. xylosoxidans* (11). This pathogen also has a remarkable ability to acquire extrinsic drug-resistance genes. In this study, the five isolates examined harbored several classes of β-lactamase genes, including class A β-lactamases (*bla*_AXC-1_, *bla*_PSE-1_, and *bla*_TEM-1_), class B β-lactamase (*bla*_NDM-1_), and class D β-lactamase (*bla*_OXA-21_). Additionally, they possessed aminoglycoside-modifying enzyme genes (*aac(6′)-Ib*, *aph (6)-Id*, *aph(3″)-Ib*, *aac(6′)-Ib-cr*, *ant(4′)-Iib*, *aph(3′)-VI*) and resistance genes for other antibiotic classes, including *tet(G*), *tet(B*), *dfrA15*, *dfrA1*, *cmx*, and *floR*.

It remains unclear whether *A. xylosoxidans* infections in humans originate from environmental reservoirs or whether this bacterium cycles between patients, hospital environments, communities, and the outdoors. The phylogenetic tree of all isolates identified as *A. xylosoxidans* (Fig. S1) revealed two distinct clades: most isolates in Clade A were from humans, whereas those in Clade B were environmental. Whole genome sequence-based identification confirmed that all isolates in Clade A were *A. xylosoxidans*, while isolates in Clade B belonged to other *Achromobacter* species, potentially including novel species. These findings suggest that *A. xylosoxidans* is a human pathogen, whereas other *Achromobacter* species are not. One case report linked a community-acquired *A. xylosoxidans* infection to the patient’s home drinking water (26). Another surveillance study in cystic fibrosis patients found *A. xylosoxidans* distributed across hospital, domestic, and outdoor environments, highlighting the exposure of immunocompromised patients to these reservoirs (7). These studies indicate a close relationship between clinical and environmental *A. xylosoxidans* isolates, although molecular epidemiological analyses were not performed. Re-evaluation using whole genome sequence-based bacterial identification in these studies is warranted. Future molecular epidemiological studies are essential to determine whether *A. xylosoxidans* populations causing human infections are distinct from or overlap with those inhabiting environmental reservoirs.

Specific isolates of *A. xylosoxidans* derived from Myanmar may spread in medical settings worldwide. The phylogenetic analysis in this study reveals that four isolates (DN2019, 41779_2, 4124363476, and MyNCGM70) were closely related; however, they were obtained from several countries and regions, including France, Canada, Russia, and Yangon, in Myanmar. Further epidemiological studies of *A. xylosoxidans* clinical isolates are necessary.

## Data Availability

The whole genome sequences of the five isolates were deposited at GenBank as following accession numbers; BAAGEZ010000001-BAAGEZ010000502 for MyNCGM70, BAAGFA010000001-BAAGFA010000801 for MyNCGM121, BAAGFB010000001-BAAGFB010000873 for MyNCGM152, BAAGFC010000001-BAAGFC010003619 for MyNCGM683 and AP038910 for MyNCGM749.

## SUPPLEMENTAL MATERIAL

Supplemental figure spectrum.00080-25-s0001.tif Figure S1.

Table S1 spectrum.00080-25-s0002.xlsx Information on 46 *A. xylosoxidans* used in the phylogenetic analysis.

Table S2 spectrum.00080-25-s0003.xlsx dDDH and ANI values of all the 46 isolates.

Table S3 spectrum.00080-25-s0004.xlsx SNP matrix among all strains of which complete genome sequences were registered in the GenBank database.

ASM does not own the copyrights to Supplemental Material that may be linked to, or accessed through, an article. The authors have granted ASM a non-exclusive, world-wide license to publish the Supplemental Material files. Please contact the corresponding author directly for reuse.
