# Supplementary figures and images for: Highly carbapenem-resistant Achromobacter xylosoxidans harboring blaNDM-1 in Myanmar
Source: Microbiol Spectr. 2025 May 19;13(7):e00080-25. doi: 10.1128/spectrum.00080-25 (PMC12210851; doi:10.1128/spectrum.00080-25)

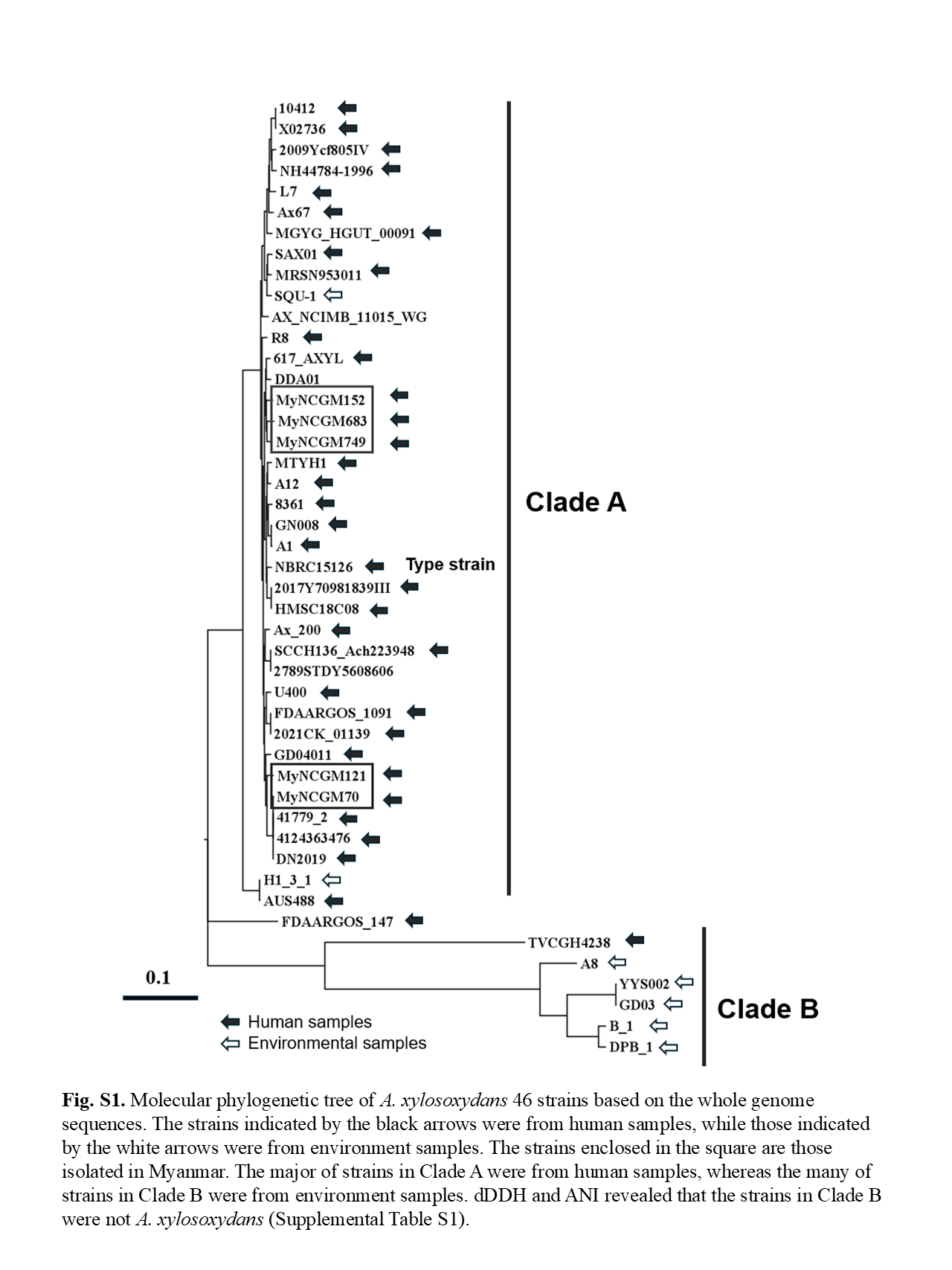

Supplement: Supplemental figure — Figure S1. [file spectrum.00080-25-s0001.tif]
